# Supplementary material for: The transcriptional response of cortical neurons to concussion reveals divergent fates after injury
Source: Nat Commun. 2025 Jan 27;16:1097. doi: 10.1038/s41467-025-56292-0 (PMC11772587; doi:10.1038/s41467-025-56292-0)
Supplement: Supplementary file 2 — Description of Additional Supplementary Files [file 41467_2025_56292_MOESM2_ESM.pdf]

Description of Additional Supplementary Files

File Name: Supplementary Data 1
Description: Number of nuclei per cluster in the snRNAseq dataset.
