# Supplementary material for: The transcriptional response of cortical neurons to concussion reveals divergent fates after injury
Source: Nat Commun. 2025 Jan 27;16:1097. doi: 10.1038/s41467-025-56292-0 (PMC11772587; doi:10.1038/s41467-025-56292-0)
Supplement: Supplementary file 4 — Reporting Summary [file 41467_2025_56292_MOESM4_ESM.pdf]

nature portfolio

Corresponding author(s): *Double-anonymous peer review submissions: write DAPR and your manuscript number here instead of author names.*

Last updated by author(s): *YYYY-MM-DD*

Reporting Summary

Nature Portfolio wishes to improve the reproducibility of the work that we publish. This form provides structure for consistency and transparency in reporting. For further information on Nature Portfolio policies, see our Editorial Policies and the Editorial Policy Checklist.

Statistics

For all statistical analyses, confirm that the following items are present in the figure legend, table legend, main text, or Methods section.

n/a	Confirmed	
☐	☒	The exact sample size (n) for each experimental group/condition, given as a discrete number and unit of measurement
☐	☒	A statement on whether measurements were taken from distinct samples or whether the same sample was measured repeatedly
☐	☒	The statistical test(s) used AND whether they are one- or two-sided<br>*Only common tests should be described solely by name; describe more complex techniques in the Methods section.*
☐	☒	A description of all covariates tested
☒	☐	A description of any assumptions or corrections, such as tests of normality and adjustment for multiple comparisons
☐	☒	A full description of the statistical parameters including central tendency (e.g. means) or other basic estimates (e.g. regression coefficient) AND variation (e.g. standard deviation) or associated estimates of uncertainty (e.g. confidence intervals)
☐	☒	For null hypothesis testing, the test statistic (e.g. F, t, r) with confidence intervals, effect sizes, degrees of freedom and P value noted<br>*Give P values as exact values whenever suitable.*
☒	☐	For Bayesian analysis, information on the choice of priors and Markov chain Monte Carlo settings
☒	☐	For hierarchical and complex designs, identification of the appropriate level for tests and full reporting of outcomes
☒	☐	Estimates of effect sizes (e.g. Cohen's d, Pearson's r), indicating how they were calculated

Our web collection on statistics for biologists contains articles on many of the points above.

Software and code

Policy information about availability of computer code

Data collection	Cell Ranger 7.0.0, 10X Genomics, Zeiss Zen Software (Blue edition), FIJI v2.3.0/1.54h, ClampEx v11.03
Data analysis	Seurat v4.1.3, RStudio v2023.12.0+369, GraphPad Prism v10, ClampFit v10.7

For manuscripts utilizing custom algorithms or software that are central to the research but not yet described in published literature, software must be made available to editors and reviewers. We strongly encourage code deposition in a community repository (e.g. GitHub). See the Nature Portfolio guidelines for submitting code & software for further information.

Data

Policy information about availability of data

All manuscripts must include a data availability statement. This statement should provide the following information, where applicable:

- Accession codes, unique identifiers, or web links for publicly available datasets
- A description of any restrictions on data availability
- For clinical datasets or third party data, please ensure that the statement adheres to our policy

The datasets generated in the current study are available from the corresponding author on reasonable request. Data will be deposited to GEO and accession codes will be available before publication.

Research involving human participants, their data, or biological material

Policy information about studies with human participants or human data. See also policy information about sex, gender (identity/presentation), and sexual orientation and race, ethnicity and racism.

Reporting on sex and gender	*Use the terms sex (biological attribute) and gender (shaped by social and cultural circumstances) carefully in order to avoid confusing both terms. Indicate if findings apply to only one sex or gender; describe whether sex and gender were considered in study design; whether sex and/or gender was determined based on self-reporting or assigned and methods used. Provide in the source data disaggregated sex and gender data, where this information has been collected, and if consent has been obtained for sharing of individual-level data; provide overall numbers in this Reporting Summary. Please state if this information has not been collected. Report sex- and gender-based analyses where performed, justify reasons for lack of sex- and gender-based analysis.*
Reporting on race, ethnicity, or other socially relevant groupings	*Please specify the socially constructed or socially relevant categorization variable(s) used in your manuscript and explain why they were used. Please note that such variables should not be used as proxies for other socially constructed/relevant variables (for example, race or ethnicity should not be used as a proxy for socioeconomic status). Provide clear definitions of the relevant terms used, how they were provided (by the participants/respondents, the researchers, or third parties), and the method(s) used to classify people into the different categories (e.g. self-report, census or administrative data, social media data, etc.) Please provide details about how you controlled for confounding variables in your analyses.*
Population characteristics	*Describe the covariate-relevant population characteristics of the human research participants (e.g. age, genotypic information, past and current diagnosis and treatment categories). If you filled out the behavioural & social sciences study design questions and have nothing to add here, write "See above."*
Recruitment	*Describe how participants were recruited. Outline any potential self-selection bias or other biases that may be present and how these are likely to impact results.*
Ethics oversight	*Identify the organization(s) that approved the study protocol.*

Note that full information on the approval of the study protocol must also be provided in the manuscript.

Field-specific reporting

Please select the one below that is the best fit for your research. If you are not sure, read the appropriate sections before making your selection.

☒ Life sciences ☐ Behavioural & social sciences ☐ Ecological, evolutionary & environmental sciences

For a reference copy of the document with all sections, see nature.com/documents/nr-reporting-summary-flat.pdf

Life sciences study design

All studies must disclose on these points even when the disclosure is negative.

Sample size	No sample-size calculation was performed. For sequencing experiments, two replicates were performed and analyzing just 1 of 2 did not dramatically change the clustering pattern nor the major conclusions. Sample sizes for histological analyses were chosen based on clear effects in wildtype animals in ipsilateral vs contralateral cortex and extrapolated to other manipulations.
Data exclusions	Animals were excluded if they were outliers in loss of righting reflex, or, in the case of Thy1-YFP animals, exhibited too-sparse labeling for certain quantifications. Cells were excluded from elecrophysiological analyses if they experience a 20% or higher increase in access resistance or high max spike rate consistent with interneurons. For sequencing experiments, outliers were identified based on number of expressed genes and mitochondrial proportions and removed from the data.
Replication	Single nucleus data were replicated twice, and resulted in consistent clustering patterns and major conclusions. Major findings, such as the differential vulnerability of layer V and layer II/III neurons, were replicated with multiple mouse strains (ie Atf3-Cre and Atf3-CreER). All immunolabeling and in situ hybridization experiments were performed across 2-4 sections per animal.
Randomization	In experiments where sham animals were used, littermates were randomized into sham or mTBI groups, maintaining roughly equal numbers of males and females per group. For other experiments, randomization was not used as all littermates with the appropriate genotype were administered mTBI.
Blinding	Wherever possible, quantification of microscopy images was performed blinded. Blinding was performed for quantifications comparing two genotypes and all quantifications performed from high magnification images. For ipsilateral vs contralateral quantifications from low-magnification images, blinding was not possible because the ipsilateral cortex is evident.

Reporting for specific materials, systems and methods

We require information from authors about some types of materials, experimental systems and methods used in many studies. Here, indicate whether each material, system or method listed is relevant to your study. If you are not sure if a list item applies to your research, read the appropriate section before selecting a response.

Materials & experimental systems

n/a	Involved in the study
	☒ Antibodies
☒	☐ Eukaryotic cell lines
☒	☐ Palaeontology and archaeology
	☒ Animals and other organisms
☒	☐ Clinical data
☒	☐ Dual use research of concern
☒	☐ Plants

Methods

n/a	Involved in the study
☒	☐ ChIP-seq
☒	☐ Flow cytometry
☒	☐ MRI-based neuroimaging

Antibodies

Antibodies used	Primary antibodies: guinea pig anti-Ankyrin-G (1:500, Synaptic Systems, 386-005), rabbit anti-ATF3 (1:500, Novus Biologicals, NBP1-85816), rat anti-CD68 (1:500, Bio-Rad, MCA1957), rat anti-CTIP2 (1:500, abcam, ab18465), mouse anti-GFAP (1:500, Sigma-Aldrich, G3893), chicken anti-GFP (1:500, Invitrogen, A10262), chicken anti-IBA1 (1:500, Synaptic Systems, 234-006), rabbit anti-phospho-cJun Ser63 (1:300, Cell Signaling Tech., 9261), rabbit anti-phospho-H2AX (1:400, Cell Signaling Tech., 2577), rabbit anti-Olig2 (1:500, Millipore, AB9610). A custom made rabbit anti-β4-Spectrin was shared by Dr. Damaris Lorenzo.
Validation	Synaptic Systems: To date nearly one fifth of our antibodies are Knock-out or Knock-down validated.<br>Novus Biologicals: ATF3 was knockout-validated.<br>Invitrogen: GFP - This Antibody was verified by Relative expression to ensure that the antibody binds to the antigen stated.<br>Cell Signaling Tech: We guarantee that our antibodies are fit for purpose by carefully tailoring the combination of validation strategies applied to each product. This means customizing our validation process according to the biological role of the target, while considering the sensitivity requirements of the downstream assay, the availability of appropriate testing models, and the relevance of each method to target investigation.<br>Validated in-house: CD68 based on co-expression with IBA1 and lysosomal morphology, CTIP2 based on expected expression pattern, GFAP, IBA1, AnkG, and Olig2 based on expected morphology.

Animals and other research organisms

Policy information about studies involving animals; ARRIVE guidelines recommended for reporting animal research, and Sex and Gender in Research

Laboratory animals	Mice; male and female; > 7w of age; Housing conditions: 12/12 hour light/dark cycle, humidity between 30-50%, temperature 72ºC.<br>Strains: Thy1-YFP, Jax Stock No. 003782; CAG-Sun1/sfGFP, Jax Stock No. 021039; Snap25-LSL-eGFP, Jax Stock No. 021879; Ai14, Jax Stock No. 007914; Rbp4-Cre, MMRRC Stock No. 037128-UCD; Ddit3 fl, Jax Stock No. 030816; Sarm1 KO, Jax Stock No. 03439; Eif2-S51A, Jax Stock No. 017601; Atf3-Cre; Atf3-CreER; DLK fl; Atf3 fl
Wild animals	The study did not involve wild animals.
Reporting on sex	For all studies, both male and female animals were used. For all analyses, potential differences between males and females were examined but not observed.
Field-collected samples	The study did not involve samples collected in the field.
Ethics oversight	All experiments involving animals were performed in accordance with animal protocol 17-003, 20-003, and 23-003 approved by the NICHD ACUC.

Note that full information on the approval of the study protocol must also be provided in the manuscript.

Plants

Seed stocks	*Report on the source of all seed stocks or other plant material used. If applicable, state the seed stock centre and catalogue number. If plant specimens were collected from the field, describe the collection location, date and sampling procedures.*
Novel plant genotypes	*Describe the methods by which all novel plant genotypes were produced. This includes those generated by transgenic approaches, gene editing, chemical/radiation-based mutagenesis and hybridization. For transgenic lines, describe the transformation method, the number of independent lines analyzed and the generation upon which experiments were performed. For gene-edited lines, describe the editor used, the endogenous sequence targeted for editing, the targeting guide RNA sequence (if applicable) and how the editor was applied.*
Authentication	*Describe any authentication procedures for each seed stock used or novel genotype generated. Describe any experiments used to assess the effect of a mutation and, where applicable, how potential secondary effects (e.g. second site T-DNA insertions, mosiacism, off-target gene editing) were examined.*
